# What are the information needs of people with dementia and their family caregivers when they are admitted to a mental health ward and do current ward patient information leaflets meet their needs?

**DOI:** 10.1111/hex.13738

**Published:** 2023-03-19

**Authors:** Emma Wolverson, Karen Harrison Dening, Zoe Gower, Pat Brown, Julie Cox, Victoria McGrath, Amy Pepper, Jane Prichard

**Affiliations:** ^1^ School of Psychology and Social Work University of Hull Hull UK; ^2^ Dementia UK London UK; ^3^ Humber Teaching NHS Foundation Trust Hull UK; ^4^ Mental Health Neuroscience University College London London UK; ^5^ Coventry and Rugby GP Alliance Coventry UK; ^6^ The Good Care Group London UK

**Keywords:** admission, dementia, mental health, patient information, psychiatric care

## Abstract

**Introduction:**

An admission to a mental health ward is an uncertain and unexpected part of a person's journey with dementia and consequently, families require information about what to expect and how to prepare. This study aimed to establish the information needs of people with dementia and their families at the point of admission to a mental health ward and to collate existing ward information leaflets to explore if they meet these information needs.

**Methods:**

This research was conducted in two parts: (1) a qualitative study using focus groups, one with people with dementia and family carers with lived experience of such an admission (*n* = 6), and another with Admiral Nurses (*n* = 6) to explore information needs at the point of admission. (2) Each National Health Service (NHS) mental health trust (*n* = 67) was asked to provide a copy of their ward information shared at admission. A total of 30 leaflets were received from 15 NHS trusts; after removing duplicates, 22 were included. A content analysis was conducted to evaluate to what extent leaflets met the information needs identified by focus groups.

**Results:**

Two main categories ‘honest, accurate and up‐to‐date information’ and ‘who is the information for’ and four subcategories were derived from focus group data. Participants felt that people with dementia and their families were likely to have different information needs. Material for people with dementia needed to be in an accessible format. Information should cover the aim of the admission, a timeline of what to expect and details about how families will be involved in care. Practical information about what to pack and ward facilities was valued. Participants spoke about the need to consider the tone of the information, given that people are likely to be distressed. The information leaflets reviewed did not meet the information needs identified by focus group participants.

**Conclusions:**

People with dementia and family carers have different information needs at the point of admission to a mental health ward. Information provided to people with dementia needs to be in an accessible format with content relevant to these needs. Wards should aim to co‐create information to ensure that they meet people's information needs.

**Patient or Public Contribution:**

This research was supported by a patient and public involvement (PPI) group of people with dementia and carers who have experience in mental health wards. The idea for the study came from the group and was motivated by their experiences. The PPI group helped with the design of the study and took part in the focus groups. The information generated has been written up in this paper, and the knowledge generated has also been used to co‐create a guide for wards on writing their information leaflets and to support the co‐creation of a public information leaflet by Dementia UK about mental health admissions for people with dementia.

## 1 INTRODUCTION

Living with dementia brings about many challenges, changes in mood and behaviour being very common and thought to be experienced by around 80% of people.^1^ There is debate around whether changes in behaviour and mood are ‘symptoms’ of dementia (sometimes referred to as noncognitive symptoms, neuropsychiatric symptoms or behavioural and psychological symptoms of dementia) or whether these changes occur as a result of unmet care and support needs.^2^ Where changes in behaviour and mood are severe, such that a person presents a risk to themselves or others, admission to a mental health ward for assessment and treatment may be considered.^3^ As such, inpatient mental health care is often a ‘last resort’ to assess and treat difficulties such as depression or anxiety and to ameliorate behaviours that are difficult to manage in community settings.^4^

There is no standard approach to providing inpatient mental health care for people with dementia. In the United Kingdom, the Royal College of Psychiatrists advocates for providing care in specialist wards just for people with dementia^4^ but recognises that many National Health Service (NHS) trusts provide older people's mental health wards that care for people with dementia alongside older people (defined typically in UK health services as those aged 65 years and above) with other mental health difficulties. Younger people with dementia may be cared for in adult mental health services (for those aged 18–65 years) or with older people with dementia.

Despite the widespread proliferation of health information about dementia, there is very little publicly available information about inpatient mental health care for people living with dementia. Health information is particularly important in mental health care because of the stigma and isolation that many people accessing services face.^5^ Older people with dementia and their older carers may have negative preconceptions about mental health care; some may have witnessed older parents with dementia being cared for within large psychiatric hospitals. Other people may have had no previous contact with mental health services and may be unclear how these facilities differ from acute hospitals or other long‐term care facilities.

Government policy in the United Kingdom^6^ emphasises the importance of providing patients with good‐quality information to encourage better participation in health care. However, research on patient information largely relates to information about medications to support compliance and raise awareness of side effects.^7^, ^8^

Health service‐related information is the most commonly reported information need for people with dementia and their families^9^; therefore, we undertook a qualitative study to address the following questions:

1. What are the information needs of people with dementia and their carers at the point of admission to a mental health ward?
2. Do patient and carer information leaflets meet the information needs of people with dementia and their family?

The intention was that the research would inform the development of a public resource for people with dementia about mental health hospital admissions and make recommendations for mental health wards about what information people with dementia and their families considered to be important, thus highlighting areas that services should consider when writing their patient information leaflets.

## 2 METHODS

### 2.1 Design

This research was conducted in two parts: (i) To understand the information needs of people with dementia and their carers at the point of admission to a mental health ward, focus groups were conducted with people with dementia, family carers and Admiral Nurses, specialists in dementia care. Categories of interest were then generated and a coding framework was developed to support the second part of the research: (ii) A content analysis to evaluate to what extent current patient information leaflets meet these information needs.

### 2.2 Recruitment and participants

#### 2.2.1 Focus groups

Two focus groups were convened using purposive sampling. The first focus group was with people with dementia and family carers. Participants were recruited from an established patient and public involvement (PPI) group of people who all have experience in care in a mental health ward for people with dementia. The PPI group is a mix of family carers (spouses and children) and people with dementia. Information about the focus group meeting was shared with group members via email. All interested potential participants registered via email and were asked to return a signed consent form. Six people out of the group of eight agreed to take part in the focus group.

The second focus group was conducted with specialist dementia nurses (Admiral Nurses) who had all provided support to people with dementia and their carers accessing mental health wards. Recruitment was via an email invite shared by Dementia UK to all Admiral Nurses and those interested in taking part were asked to contact the researcher. Six Admiral Nurses agreed to take part.

This yielded an overall sample size of 12 participants.

#### 2.2.2 Ward information leaflets

Each of the 67 mental health trusts in the United Kingdom was contacted via email by one author (Z. G.) and asked to share a copy of their patient information leaflet that would be given to a person with dementia and/or their family members when they were admitted to a mental health ward.

### 2.3 Procedures and data collection

#### 2.3.1 Focus groups

To sensitise participants before the focus groups and to help initiate discussions, participants were emailed copies of five different ward information leaflets and asked to consider what they liked or did not like about each. Five leaflets were shared so as not to overwhelm participants but to provide enough examples to allow for comparison. For pragmatic purposes, the five leaflets selected were simply the first shared with the team following the requests to NHS sites. Focus groups were conducted by the first two authors (E. W. and K. H.‐D.). E. W. (DClinPsy) is a Clinical Psychologist and K. H.‐D. (PhD) is a nurse. Both are female researchers with a research and clinical expertise in mental health care for people with dementia and with experience in conducting focus groups. E. W. chairs the PPI group and so had an established relationship with participants of focus group 1. K. H.‐D. is an Admiral Nurse and works for Dementia UK, the charity that supports and develops Admiral Nurses. She had established relationships with participants in focus group 2. Therefore, both researchers can be considered insiders. Both authors believed that admission to a mental health ward was a stressful experience for people with dementia and their families and that support and information at this time are crucial. Participants were aware that the researchers had a wide range of experience in this care setting and were motivated to try and improve care and support during such an admission. During each focus group, field notes were made by the first author (E. W.). To guide the focus group discussions, a topic list was developed based on the literature^9^ and through discussions amongst the research team. The topic list covered (1) what participants liked about the information leaflets, (2) anything participants did not like, (3) what information was useful at the point of admission in their experience, (4) any information participants felt was missing from these leaflets that might be important, (5) what participants thought of the format of the leaflet and (6) a chance for any other comments or reflections. Both focus groups took place online and lasted between 50 and 60 min. Each focus group discussion was video‐recorded and transcribed verbatim.

#### 2.3.2 Ward information leaflets

Leaflets were collected from July to September 2022. Organisations were asked for leaflets they currently provide to patients and/or families. Leaflets were received and assessed against inclusion and exclusion criteria:

1. Written in the English language.
2. Given to patients and/or families when a person with dementia is admitted to a mental health ward.
3. Format a leaflet or a brochure.
4. Information currently used, irrespective of the date published.

Leaflets were excluded if

1. Patients and families would not access them at the first point of contact with the ward.
2. They were in the format of videos or posters. Videos were not included as these were very light on content and were typically used to supplement information leaflets. They were used to introduce the team and showcase feedback from carers rather than impart information.
3. They were on specific treatments or medications.

### 2.4 Analysis

#### 2.4.1 Focus groups

An inductive content analysis of focus group transcripts was performed by the two lead researchers E. W. and K. H.‐D.; the method was chosen because the inductive content analysis is particularly useful when there is little existing research in the area, when questions asked are specific and direct and when the research is aiming for a practical application of the findings.^10^ Inductive content analysis involves iterative coding. Initially, both researchers read and familiarised themselves with the transcripts. Both researchers agreed that saturation had been reached, noticing the same codes repeatedly across both focus groups. The first‐round coding was undertaken independently, and then the two researchers compared the texts that were assigned to codes to reach a consensus on broad code definitions. Second‐round coding involved the development of subcategories that were refined and collapsed through discussions and close reading and rereading of the transcripts, resulting in a coding tree. Finally, the codes were synthesised and interpreted into a narrative to provide an answer to the research question. The researchers E. W. and K. H.‐D. met regularly to reflect on the data analysis. To ensure the trustworthiness of the data, field notes and video recordings of meetings held on Microsoft Teams were used to triangulate the data.

#### 2.4.2 Ward information leaflets

The data generated from the focus groups formed the basis of a coding framework; this contained 17 separate coding categories, which were grouped together into three broad themes: basic ward information, treatment and care and practicalities. The units of observation were individual leaflets and the units of analysis were sentences. Manifest content analysis^11^ was conducted by counting the number of times a category of interest was stated in the leaflet; this allowed us to determine whether certain types of information were more prevalent than others in information leaflets.

## 3 RESULTS

After an iterative open and coding process, two main categories and four subcategories were derived from the data.

### 3.1 Main category: Honest, accurate and up‐to‐date information

All participants spoke about the importance of honest, accurate and up‐to‐date information. Information should not make false promises such as referring to recovery or returning home, which for many patients, may not be an option. Out‐of‐date information was worse than getting none at all; some leaflets had been created 10 years ago and participants expected information would be outdated and could cause confusion. Participants felt that information should relate to (1) treatment and care and (2) practicalities.

### 3.2 Subcategory: Treatment and care

A clear statement of the aim of admission was seen as essential by both focus groups, with family caregivers stating that this was never explained:

We were not clear going into it. What time frame? What purpose? (PPI Focus Group, Participant 1)

I didn't know what I was going to. I did not have time to research it. The only information I had was what one of the mental health nurses had given me. My previous experience of mental health inpatient units was with my gran and that was horrendous. (PPI Focus Group, Participant 5)

It was unclear to some PPI focus group participants what treatment entailed in a mental health ward and so information on the ‘use of drugs or other therapies’ was important.

Participants from both focus groups liked the inclusion of a timeline or flow chart;

‘a structured understanding of what happens when, so a timeline, I didn't really understand what happened’ (PPI Focus Group, Participant 2).

Key meetings and decision points should be explained to families in advance. Clear guidance on how the ward would communicate and involve families (not just visiting guidance) was viewed as important.

Information on the mental health act and the rights of the person and their family should be provided;

‘these are your rights, you've got every right to ask rather than feeling that you've gotta have permission. Shouldn't that be, you know, a core part of any leaflet?’ (Admiral Nurse Focus Group, Participant 3).

Some leaflets included a section detailing the ward team and the various staff members and their roles. There were mixed views on how much detail should be provided about staff roles; some PPI participants found this helpful, whereas Admiral Nurses worried that this would become quickly out of date and could be overwhelming. In addition, participants of both groups wanted to know who else would be on the ward; this often related to concerns about the safety and privacy of the person with dementia and a clear statement to this effect could be reassuring.

Given the high levels of physical comorbidity in this patient group, some reference to how the ward would manage physical health conditions was also seen as important. Three family carers had relatives who came to the end of their life during their mental health admission and suggested that information leaflets reflect that families can talk to ward staff about future care wishes in respect of end of life.

### 3.3 Subcategory: Practicalities

Practical information on what to pack and what technology devices could be brought onto the ward was seen as important as many older people were now both familiar and reliant on such items. Information about meals, laundry, parking or public transport and how to contact the ward was all viewed as essential. Details such as examples of meals, menus or social and therapeutic activities were also welcomed as people with dementia were thought to benefit from more concrete information. Some leaflets used images, for example, in showing a picture of a meal as a food option. However, both groups felt that this required careful consideration to ensure that good‐quality images were used but also that these were inclusive of dietary options for all potential patients.

Information about how wards support spiritual needs and the needs of those for whom English is not their first language was also felt to be important.

Information about the environment and facilities was found to be helpful and participants from both groups wanted to see photographs of the actual ward environment but felt that these should not ‘dressed’ or ‘staged’;

‘the images on the [names ward leaflet] I thought were really helpful, especially when you can't visit. So maybe for families that live a long way away or particularly through the pandemic, just to actually be able to visualise where your loved one is. So what do the rooms look like? You know, where are they going to have their dinner, etcetera’ (PPI Focus Group, Participant 6).

Admiral Nurse participants felt that there was a balance to be struck with the use of images of the ward in that they were accurate in their representation of the ward. They reflected on their experiences of environments often seeming austere or lacking in home comfort, largely due to the risks that certain objects might present to patients, such as glazed pictures, vases, and so forth.

### 3.4 Main category: Who is the information for?

#### 3.4.1 Subcode: Audience

Participants of both groups discussed who wards should direct their information leaflets to, given that there were two very distinct groups: patients and family carers.

Some of them [leaflets] were a little bit confusing because some of them sort of flitted between addressing the person who'd been admitted and addressing the relative like, you know, sort of almost paragraph to paragraph. (Admiral Nurse Focus Group, Participant 2)

Do we need two versions, one for the person with dementia and one for the carer? (PPI Focus Group, Participant 3)

People with dementia often need information in an accessible format that takes into account sensory needs and considers the length of the document. Admiral Nurse participants felt that people with dementia might require different information than that of their family carers and that the content of any leaflet should be adapted for the audience. An example given was that people with dementia might want to know who is going to care for them, how to access the gardens and how to contact their family. Family carers might need to know about parking, laundry and what support is available for them.

The use of jargon should be avoided and participants in both groups felt that prior knowledge of mental health care or systems should be assumed. There are a lot of rules imposed on patients and families in mental health care and particularly in the care of people who are detained under the Mental Health Act. Admiral Nurse participants felt that rather than listing rules, clear explanations needed to be given, for example, why doors are locked, why permissions to go on leave are needed. It was noted that some leaflets contained language that is no longer considered politically correct or not used in dementia care (e.g., ‘dementia patients’) and did not comply with language guidelines in dementia.

The tone of the information was seen to set a standard for communication and expectations for what the ward would be like. Some information leaflets were seen as ‘cold and clinical’ and felt unwelcoming and adding to a sense of fear. Some wards spoke about delivering relationship‐centred or person‐centred dementia care, but this was not supported in how they communicated about the ward or people with dementia;

‘If you're saying you're treating people as equals, for example, it needs to be written in a way that that does that and communicates that. And I think there's a danger, isn't there, with the welcome bit and I think some of the leaflets, when you look at them, are very unwelcoming’ (Admiral Nurse Focus Group, Participant 1).

The welcome in particular needed careful thought as many people admitted and their family members did not wish for such an admission;

‘you don't want to visit and you don't want your loved one to be there’ (PPI Focus Group, Participant 4).

It was felt that leaflets should be honest;

‘acknowledge that people will be there because they've had difficult experiences’ (PPI Focus Group, Participant 3).

Part of considering the audience was also thinking about the use of images carefully; clip art and cartoons were seen to be in bad taste. The sole use of images portraying older people was disliked by those with young‐onset dementia and felt not to reflect the entire population of people admitted with dementia

I think people who get, who are sectioned are physically quite active and generally on the younger end and just very distressed. (PPI Focus Group, Participant 1)

The participants felt that leaflets needed to be more widely available and shared with care homes, GPs and hospitals as many of these services had been ill‐informed about mental health wards and had not been able to offer any advice or sources of information and are ‘seriously lacking the knowledge’ (PPI Focus Group, Participant 5).

#### 3.4.2 Subcode: Timing

The timing of the information shared was also considered to be important. Participants of both groups spoke about wards needing to build trust and offering reassurance rather than listing rules. Given that people are likely to be distressed at the point of admission, families felt some information such as details of foot care could be shared at a later date when they were in more of a position to take such things on board.

We sort of need to think about who's reading this leaflet when, you know, when you are reading it when you're in the middle of a crisis and you know you've just had somebody say, right, we're going to admit them and you've just got to try and understand what's going. (PPI Focus Group, Participant 4)

### 3.5 Analysis of ward leaflets

The publication dates of leaflet in the analysis ranged from 2012 to 2022. Many leaflets (*n* = 11) were not dated and may have been produced outside this date range. Sixty‐six NHS trusts were contacted; 45 did not reply. Of those that did reply, 3 replied stating that they had no leaflets, 2 stated that they share information in a video format, 2 stated that they share both videos and leaflets and 15 trusts replied, sharing patient information leaflets. Thirty‐seven leaflets were received and assessed against inclusion and exclusion criteria. A total of 30 ward leaflets from 15 different NHS trusts fulfilled the inclusion criteria. Six trusts had standardised leaflets that were used for each of their wards; we identified these leaflets as duplicates and only included them individually in the analysis if there were differences in text and appearance. Therefore, 22 leaflets were included in the final study.

#### 3.5.1 Basic ward information

Of the 22 leaflets included in the analysis, 9 were written for patients, 2 for patients and carers, 4 exclusively for carers and in 7, it was unclear who the intended audience was. Only one trust shared two leaflets for a ward: one written for carers and one written for people with dementia.

Contact details for the ward were included in 19 leaflets, 13 described the roles of staff and 20 gave a description of the ward environment and its facilities, although the level of detail provided varied significantly. Pictures of the ward were provided in 12 leaflets; most showed a picture of the outside aspect of the ward, with only one providing photographs of the internal ward environment.

The aim of the admission was described in 12 leaflets (see Table 1). Assessment and treatment were commonly described as the main aims of the admission. Intended outcomes of the admission included promoting recovery, facilitating a return home, to a care home or community setting, promoting well‐being, improve quality of life and independence. Three leaflets described the importance of partnership working with families and people with dementia, one referred to specialist staff and one a specialist environment.

**Table 1:** Aim of admission is described in patient information leaflets.

Stated ward aims
To assess and treat males over the age of 65 years who are experiencing severe mental health difficulties and living with dementia.
We aim to provide individual care and treatment, working both with patients and carers, to promote recovery and your return home.
Our aim is to work in partnership with patients and carers to promote the mental, physical and spiritual well‐being of people who access the help and support of the Older People's Mental Health Services.
The purpose of admission to the ward is to enable safe and timely assessment and treatment by specialist staff and the development of a long‐term plan of care. The aim is to return you to a community setting as quickly and effectively as possible.
Our main aims are to help improve health and well‐being, increase confidence and independence in day‐to‐day living skills and for your relative/friend to return to a good quality of life after they have left the hospital.
We aim to provide a full psychological and physical assessment to enhance individualised and person‐centred care.
We aim to work together with you to help you manage your needs so that you can move forward in your life with the support you need in the most acceptable way for you.
Our goal is always to treat each person as an individual and support independence, maintain contact with loved ones and promote enjoyment in activities and the best quality of life possible.
We aim to assist recovery or rehabilitate to allow them to move back into the community. This may be to their own home, a residential setting or a nursing home.
Our role is to offer a holistic assessment and review of the person's needs.
We provide a safe and supportive environment to help you get better and return to the community, where you may continue your recovery.

Eight leaflets made reference to the mental health act and to the fact that many people may not have consented to their admission.

#### 3.5.2 Treatment and care

Details of the interventions offered during admission and what ‘treatment’ would involve were very limited in all information leaflets reviewed. Eleven leaflets (50%) referred to ‘therapy’ being offered as part of admission including occupational therapy (*N* = 5), psychological therapy (*N* = 5) and specific interventions including cognitive stimulation therapy (*N* = 3) and life story work (*N* = 4). The provision of ‘therapeutic activities’ was referred to in 12 (55%) leaflets and included a range of activities (see Table 2). The use and review of medications to treat distress were referred to in nine leaflets.

**Table 2:** Activities offered during a mental health admission.

Health and fitness classes
Social groups
Baking and cooking groups
Reminiscence activities
Games and quizzes
Singing/music therapy
Bowling
Walking
Gardening
Pets as therapy
Sensory room
Newspaper review
Bingo
Massage therapy
Creative groups
Reading and magazine groups

The treatment of people's physical health during their admission was referred to in 16 (73%) leaflets, with reference to wards reviewing physical health as part of their overall assessment. The risk of falls was mentioned in four (18%) leaflets. None of the leaflets made reference to what would happen if a person needed hospital admission to an acute hospital during their admission and none of the leaflets made reference to end‐of‐life care.

The provision of support to family caregivers during admission was referred to in 11 (50%) leaflets. Such support included a referral to Recovery College courses (*N* = 1), the provision of information about dementia (books on prescription) (*N* = 1), chaplaincy support (*N* = 2), social events organised for carers (*N* = 1), a referral for a carers assessment (*N* = 4), referral for carers' passport (*N* = 1), ward‐based group carer support sessions (*N* = 1), one‐to‐one support provided by ward staff (*N* = 4) and involvement at carer forums (*N* = 1). A number of these leaflets also included links and contact numbers for carer agencies and support services for people to self‐refer to.

Information about discharge was provided in 15 (68%) leaflets and some wards offered a separate leaflet on preparing for discharge and supporting discharge.

#### 3.5.3 Practicalities

Leaflets were largely focused on providing practical information relating to admission as shown in Table 3. The most commonly covered topic was visiting; of these, 19 (86%) leaflets reported set visiting hours, whereas 3 (14%) referred to signing John's campaign pledge stating that they were committed to involving family carers and allowed visiting out of any restricted hours.

**Table 3:** The practical information is provided in information leaflets.

Topic	Meals	Clothing	Privacy	Visiting	Safety	Spirituality	Translation/interpreter services
% of leaflets containing information	82	86	82	100	73	77	73

Ward safety was mentioned in 16 (73%) leaflets with reference to items not to bring onto the ward, controlled access to the ward, visiting restrictions, observations, use of restraint, sexual safety, CCTV, falls, medication administration and safeguarding issues. Details on translation or interpreter services were referred to in 16 (73%) leaflets and typically asked people to request services if required. Spiritual support was referred to in 17 (77%) leaflets.

## 4 DISCUSSION

An admission to a mental health ward for a person with dementia usually occurs at a time of crisis and can be an incredibly distressing time for the person and their family. The provision of timely and up‐to‐date information is important, but it must take into account the emotional distress of the people reading it. As such, the tone and language matter as much as the content. Research indicates that communication between families and staff on mental health wards can be strained^12^ and the provision of accurate information with clear roles and responsibilities on the part of the ward and its staff as well as expectations of family carers might help to build open communication. Moreover, information leaflets provided to carers should recognise the strain that they are under and outline what support is available to them, their rights and how they can continue to stay involved in their relatives care.

As people with dementia have different information needs for their families, we propose that mental health wards should produce separate information for people with dementia and carers. Even though some of the information leaflets reviewed were targeted at the person with dementia, none of them were presented in an appropriate accessible format.^13^ Furthermore, language guidelines in relation to dementia should be adhered to.^14^ Some trusts shared welcome videos (which were not the focus of this work) and such a hybrid approach to sharing information could be useful. However, the videos that we received were often too long for people with dementia and none had any subtitles.

Overall, the information leaflets reviewed did not meet the information needs of people with dementia and their carers; therefore, in collaboration with the PPI group, we have created a guide for wards on producing their patients' information (see Supporting Information: Appendix 1). Undoubtedly, the best way to ensure that leaflets are written in a way that is meaningful and understandable to people with dementia and their carers is to co‐create them. Co‐design is a well‐established approach in research and service design practice and is particularly valuable in dementia care as it can offer novel ways of complementing existing approaches to care to improve their quality of life.^15^, ^16^ People with dementia have co‐designed research trials,^15^ clinical services^17^ and healthcare technologies.^18^ Co‐creation may be particularly useful in the mental health context because co‐design uses the expertise of those with lived experiences of services (both consumers and carers) and provocateurs (curious questioners) to understand a ‘problem’ and develop innovative strategies to address it. It relies on the creation of a safe environment where power imbalances are acknowledged and mitigated, and decisions are made collaboratively.^19^ The challenge here is that research demonstrates that people with dementia who are admitted to mental health wards are often in the advanced stages of their dementia and are very unwell^20^; it may be that wards have to engage people much earlier in their dementia and support them to think about their future wishes. However, this may be difficult as it is possible that people might not want to consider or might not be aware of mental health wards.

This study has several limitations. Firstly, the focus groups were small in size and contained more carers and professionals than people with dementia. Not all trusts replied to our information requests, so it is possible that further examples of ward information leaflets are available and were not included in this analysis. The strength of this study is that it is the first of its kind in matching the information needs of family carers and people with dementia at what is for many a point of crisis in their dementia journey.

## 5 CONCLUSION

People with dementia and carers are likely to have different information needs at the point of admission to a mental health ward. The information provided to people with dementia needs to be in an accessible format. Current ward information leaflets may not meet the needs of carers or people with dementia. Wards should try to co‐create information to ensure that they meet people's information needs.

## AUTHOR CONTRIBUTIONS

Emma Wolverson and Karen Harrison‐Dening designed the study, conducted the focus groups and were responsible for the data analysis of the focus groups. Zoe Gower was responsible for collecting the leaflets and assisted Emma Wolverson in the content analysis of the leaflets. Emma Wolverson obtained ethical approval for the study and wrote the manuscript. Pat Brown, Julie Cox, Victoria McGrath, Amy Pepper and Jane Prichard assisted in the analysis of the focus group data, in collating the guidance for wards and provided support in improving and editing early drafts of the manuscript.

## CONFLICT OF INTEREST STATEMENT

The authors declare no conflict of interest.

## ETHICS STATEMENT

Ethical approval was given by the University of Hull Faculty of Health Science Research Ethics Committee.

## Supporting information

Supporting information.

## Data Availability

The leaflets analysed in the study are public resources and available from National Health Service trusts. The focus group data are not publicly available.
