# Supplementary material for: What are the information needs of people with dementia and their family caregivers when they are admitted to a mental health ward and do current ward patient information leaflets meet their needs?
Source: Health Expect. 2023 Mar 19;26(3):1227–35. doi: 10.1111/hex.13738 (PMC10154859; doi:10.1111/hex.13738)
Supplement: Supplementary file 1 — Supporting information. [file HEX-26--s001.docx]

**Appendix One: Inpatient Mental Health Wards for People with Dementia**

**A staff guide for writing a ward leaflet**

1. **Introduction**

**Who is this guide for?**

This guide is written for staff who work within inpatient mental health wards for people with dementia. The aim is to provide some ideas and things to consider when creating your ward information packs/ leaflets for people with dementia who may be admitted to your ward(s) and their families.

Being admitted to a mental health ward can be a very frightening and distressing experience for a person and their family, these leaflets can be an important way for people to find out what to expect and they can set the tone for good communication with the ward.

**How was it created?**

This guide has been co-created with people living with dementia and their family carers who have experience of mental health inpatient care, with Admiral Nurses, researchers and health care professionals.

1. **Getting the basics established**

| **Key Points:**   - People with Dementia and family carers have different information and accessibility needs - Co-create your leaflets in collaboration with people with dementia and carers to check the content, tone, language and images |
| --- |

**Who is the leaflet for?**

Be clear who the intended audience is for your leaflet. A lot of the leaflets we reviewed didn’t specify who they were for. Family carers have different information needs to people with dementia who are on the ward, so each require a bespoke information leaflet

Where a leaflet is being written for a person with dementia it may require the format to be written in a more accessible and easy to read version.

**When is it being given out?**

When a person is first admitted family members often had very practical questions about clothing, washing, meals and how to contact the ward, in addition to other questions e.g. about the purpose of the admission, how long will my relative be an inpatient, what happens in an assessment? An overview of what to expect when can be useful to families.

However, too much information at once could be overwhelming; for example, information about discharge planning might be less important at this point. You might want to consider a series of shorter information leaflets that are given out at different times over the course of an admission. Some people might want all the leaflets up front, and others might only want the information on a staged or need to know basis.

**Where do you put the leaflet?**

Many wards struggled to find leaflets when we asked for them. Some wards post these to everyone on admission, some were in waiting rooms and some were on websites. Consider how accessible your leaflets are; how will people find them or at what stage will you give them out?

Can care homes and AMPS give families accurate information about the wards? Maybe they need copies of information too.

1. **Language and tone**

**Welcome pack or ward information?**

Think carefully about the tone of your welcome. The people reading it are likely to be scared, angry and upset. If the information leaflet is given at the point of admission to your ward, most people have been or are in crisis - you might want to acknowledge this distress. One member of our expert group with dementia disliked the “welcome” section as pointing out its not somewhere you want to be or chose to be. Family members told us they often felt that they had failed their relative, they wanted reassurance that their views and opinions still mattered and that they could continue to be involved in their relative’s care if they wished.

**Language**

Consider the language you use, for example, avoid the use of ‘dementia patients’ as old fashioned, institutional type language. When developing any leaflet aimed at people with dementia use the DEEP guidelines on the most appropriate language and style.

Avoid terms like “difficult behaviors” and “challenging behaviors”; “distress” or “unmet needs” are better terms. We also suggest the term “loved one” is avoided when referring to a relative, given that relationships may have become very strained or even broken down prior to admission.

Avoid jargon and acronyms. Similarly, some terms and expressions used may have meaning for the ward staff but none for the person with dementia or their family carer. What does ‘we aim to provide a full psychological and physical assessment to enhance individualised and person centred care’ mean to the non-professional audience?

Avoid using any academic references unless they are easy to read or summarised or are available on an open access basis.

It is important that your information is informative yet accessible without being at such a low level that it comes over as patronizing or so technical that it renders itself useless.

**Top tips:**

- **Give explanations:** several leaflets had long lists of rules and don’ts (such as don’t bring family photos or don’t visit at mealtimes) they made the ward sound very strict and quite scary. Please always explain rules.
- **Avoid the expert trap:** Don’t set yourselves up as the experts with all the answers.
- **Don’t make false promises:** be careful when using words like “recovery”. Some leaflets spoke of people going on leave or being discharged home which may not be the reality for everyone.
- **Diversity:** Make sure that information about interpreter services is clearly visible; people should not be left hunting for this information.
- **Disability compliance:** many prospective patients and family carers may have sight problems or other reasons for having difficulty in being able to use an information leaflet. Consider font sizes, colour, images, etc.
- **Date and date for review:** some leaflets we found were nearly 15 years old. Many didn’t have a date on them to indicate when they were created. All leaflets should be dated and marked with a date for review. If you are including information about your staff team it is important to ensure that your leaflet is updated regularly to factor in staff turn-over.

1. **Images**

Very few of the leaflets that we reviewed had any images. Pictures of the internal ward environment would be valuable. Seeing images of rooms and gardens helped people who were unable to visit or lived some distance away to visualize the ward. These should be real images not “dressed” for the camera.

Be careful when using cartoon images as some find these insulting. Remember too that there are younger people with dementia on mental health wards and ensure images reflect this and are not just of older people with dementia.

1. **What information to include**

Having reviewed a number of leaflets and reflecting on their own experiences, here is some of the information our group felt it would be valuable to include in the ward information:

**For families**

- **Who’s Who**: Introduce the staff team and their roles
- **Walk through of what happens:** from admission to discharge, flow charts were very helpful
- **Family involvement**: how you will support, involve and communicate with families to ensure they are kept updated? How can families get to the ward - practical information like transport links and parking were welcomed. Consider enabling visiting rather than restricting it; include information about John’s Campaign.
- **Visiting**: when, how to arrange, where, can children and/or pets attend?
- **Who to contact and when**: phone numbers and names. Does the person with dementia and their family members have a dedicated nurse allocated to them? Does the ward manager hold a surgery for families to aid communication?
- **Purpose of the admission:** why are people admitted to your ward? You may not be able to cite all situations but it would be useful to give some detail of some of the more common reasons. Who else is likely to be there (is it a mixed ward, a specialist dementia ward?)
- **What to pack**: Information about what to pack was important and families said it was important to point out that things will get lost.
- **The risks**: The risks of an admission was also something to consider adding, although again tone is important and it is important not to add to peoples worries.
- **Explain all mental health terminology and processes**: Don’t assume any prior knowledge or experience of mental health services in a family. Many families did not know the difference in a mental health hospital to an acute/general hospital. Some may hold negative associations with mental health hospitals and still think in terms of the old asylum type hospital. A guide to the Mental Health Act relating to the admission – common terms and language used, consider a glossary or easy read document explaining this.
- **Illness**: What happens if people become physically unwell during their admission to the mental health ward?
- **Examples menus**: Food was a big worry
- **Discharge**: Some information about how discharge from the ward will be planned, family members said they wanted reassurance that someone would not just be sent home like they might be from a general hospital.
- **Timescales:** Explaining roughly how long a person may be on the ward for, (whilst clearly difficult to give exact timescales) was considered useful. Many people believed that someone would be sent home after the 28 days were up. Others considered that timescales would be like those in a general hospital. A ward average length of stay might give people some indication of what to expect.

**For people with dementia:**

Here are some questions people with dementia might value the answer to:

- Will I have my own bedroom?
- Where is the nearest toilet?
- Who’s in charge?
- Will I be able to get out at times, have access to a garden?
- Can I use devices on the ward, such as, iPads, mobile phones, smart watches?
- How long am I likely to be in hospital for?
- What happens on the ward?
- What is there to do on the ward?
- How will I get my medication?
- Can I smoke?
- How will I get my meals?

1. **Format/ Style**

Most of the information we reviewed was in a written format. Some wards had video welcomes and ward tours (see for example: [Our Services | Herefordshire and Worcestershire Health and Care NHS Trust (hacw.nhs.uk)](https://www.hacw.nhs.uk/services/service/meadow-ward-new-haven-32/)). Some people really like videos but agreed these need to be short if they are for people with dementia and should have subtitles.

In terms of the presentation of information we like:

- Q&A or frequently asked questions formats
- Information that led people through in a logical order
- Flow chats of what happens and when

Remember every dementia is different and peoples needs will be different make sure you can present information in a range of formats.

1. **Useful Resources**

Guide to making accessible leaflets:

<http://www.plainenglish.co.uk/services/crystal-mark/7-the-crystal-mark-standard.html>

Easy read mental health act information:

<https://www.nhs.uk/mental-health/social-care-and-your-rights/mental-health-and-the-law/mental-health-act/>

<https://www.mind.org.uk/information-support/legal-rights/mental-health-act-1983/about-the-mha-1983/>

DEEP guidelines [Words Matter (dementiaaction.org.uk)](https://www.dementiaaction.org.uk/dementiawords)

John’s Campaign [John's Campaign (johnscampaign.org.uk)](https://johnscampaign.org.uk/)

1. **Checklist**

This checklist has been created to help you review your ward information

| **Things to consider** | **Yes/No (if not rationale for why not)** |
| --- | --- |
| Have you clearly stated who the leaflet is for and written for this audience? |  |
| Have you clarified when the leaflet going to be given out? |  |
| Where will the leaflet be available? |  |
| Have people with dementia / family carers been involved in writing or checked the information? |  |
| Have you given explanations for ‘rules’? |  |
| Have you made it clear that versions are available in other languages? |  |
| Have you sought guidance on disability compliance? |  |
| Have you included date created and date for review? |  |
| Have you included images of inside your ward? |  |
| Have you explained who works on the ward and their roles? |  |
| Have you explained who to contact and when? |  |
| Have you explained how families will be involved in their relatives care and kept up to date? |  |
| Is the purpose of the ward clearly stated in plain English? |  |
| **FOR FAMILIES** | |
| Have you provided guidance on what to pack? |  |
| Have your clearly stated the risks of an admission? |  |
| Have you described all mental health terminology or included a glossary? |  |
| Have you explained what will happen if people become unwell during their admission? |  |
| Have you provided information about meals? |  |
| Have you provided some timescales, such as an average length of stay for your ward to manage people’s expectations? |  |
| **FOR PEOPLE WITH DEMENTIA** | |
| Have you considered how to make the information accessible to people with dementia? |  |
| Have you provided details about bedrooms and bathrooms? Including photos. |  |
| Have you explained who is in charge and how to arrange to speak with them? |  |
| Have you provided guidance on use of devices such as phones, iPads, smart watches? |  |
| Have you explained what happens on a daily basis on the ward? |  |
| Have you detailed what there is to do on the ward? |  |
| Have you explained smoking rules? |  |
| Have you provided information about how people will get their medication? |  |
